# Dynamic Structure of Joint-Action Stimulus-Response Activity

**DOI:** 10.1371/journal.pone.0089032

**Published:** 2014-02-18

**Authors:** MaryLauren Malone, Ramon D. Castillo, Heidi Kloos, John G. Holden, Michael J. Richardson

**Affiliations:** 1 Center for Cognition, Action & Perception, Department of Psychology, University of Cincinnati, Cincinnati, Ohio, United States of America; 2 Universidad de Talca, Talca, Chile; University of California, Merced, United States of America

## Abstract

The mere presence of a co-actor can influence an individual’s response behavior. For instance, a social Simon effect has been observed when two individuals perform a Go/No-Go response to one of two stimuli in the presence of each other, but not when they perform the same task alone. Such effects are argued to provide evidence that individuals co-represent the task goals and the to-be-performed actions of a co-actor. Motivated by the complex-systems approach, the present study was designed to investigate an alternative hypothesis — that such joint-action effects are due to a dynamical (time-evolving) interpersonal coupling that operates to perturb the behavior of socially situated actors. To investigate this possibility, participants performed a standard Go/No-Go Simon task in joint and individual conditions. The dynamic structure of recorded reaction times was examined using fractal statistics and instantaneous cross-correlation. Consistent with our hypothesis that participants responding in a shared space would become behaviorally coupled, the analyses revealed that reaction times in the joint condition displayed decreased fractal structure (indicative of interpersonal perturbation processes modulating ongoing participant behavior) compared to the individual condition, and were more correlated across a range of time-scales compared to the reaction times of pseudo-pair controls. Collectively, the findings imply that dynamic processes might underlie social stimulus-response compatibility effects and shape joint cognitive processes in general.

## Introduction

Social interaction is a hallmark of everyday activity and shapes many aspects of human behavior. Examples include people playing a team sport, co-workers problem solving or brainstorming, a parent helping a child get dressed, a couple washing dishes together, or two friends carrying a heavy item up a flight of stairs. In each of these cases, a form of cooperation emerges between the actors involved, such that the physical and cognitive activity of each individual becomes coordinated with the physical and cognitive activity of the joint actors around them. Interestingly, such joint-action coordination need not emerge intentionally and often occurs unintentionally, even when no explicit coordination is required. For example, an individual’s behavioral movements or responses to environmental stimuli can be changed or altered (both negatively and positively) by the presence of another actor, even when that co-present actor is completing a separate behavioral task (for reviews see e.g., [1], [2], [3]). Most recently, this latter form of joint-action coordination has been demonstrated in research examining the response behavior of individuals completing social or joint-action stimulus-response compatibility tasks (see e.g., [4], [5]). The present study was designed to further investigate the dynamics of such joint-action stimulus-response compatibility effects and to determine the degree to which unintentional coordination phenomena might be the result of an interpersonal perturbation process.

### Joint Stimulus-Response Compatibility (JSRC)

Over the past decade, a growing amount of research has been conducted investigating joint-action via so-called ‘go/no-go tasks’ (e.g., [6], [7], [8]). In such tasks, participants are instructed to ‘go’ when given a certain stimulus context (e.g., when they are presented with a red stimulus), and to ‘not go’ when given the alternative (e.g., a blue stimulus image). The compatibility aspect of these experiments lies in the spatial orientation of the stimulus relative to the location of the responding individual. For instance, if a stimulus is presented on the same side of a display with respect to where a participant is seated (e.g., on the left), the response is deemed ‘compatible’. Alternatively, if a stimulus is presented on the opposite side of a display screen with respect to where a participant is seated (e.g., right), the response is deemed ‘incompatible'.

To examine the effects of such stimulus-response mappings in a joint-action setting, reaction times (RTs) are compared between two conditions: one in which the participant sits on one side of the display screen and responds alone to one stimulus type (the individual condition), and another where the task requirements are exactly the same except that another participant, seated on the opposite side of the display screen, responds to the alternative stimulus (the joint condition). The general finding is that even though participants in the joint condition are performing the exact same task as in the individual condition, a greater stimulus-response compatibility effect exists when two people are completing the task in one another’s presence compared to when they complete the task alone. Specifically, incompatible responses are significantly slower than compatible responses in joint conditions, but not for individual conditions.

These findings are generally taken as evidence for the co-representation of action goals during a joint-action setting, whereby actors form a shared representation of the collective task goal. That is, individuals are thought to mentally represent the actions of their co-actor and integrate them into their own action planning. This co-representation or action integration therefore results in slower RTs for incompatible stimulus situations compared to compatible stimulus situations. When completing the task alone, however, no such integration or co-representation occurs, and thus the spatial compatibility of the stimulus has little or no effect.

The JSRC effect has been observed across a wide range of stimulus and response manipulations, including hand posture [9], non-biological response mechanisms [10], orthogonality of stimulus location [11], [12], and auditory stimuli [13]. It is also known to be influenced by various social psychological variables, such as the facial features of a co-actor, and task-sharing paradigms [14], [15].

JSRC effects also appear to suggest that knowing what another person’s task is during joint-action is the means by which an individual can understand others’ action intentions and point to *shared representations* as the causal basis of this integration or modulation process. However, recent research has demonstrated that the JSRC effect can be elicited not only when participants share the same task space as another person, but also by the presence of any dynamic stimuli [16]. This latter finding highlights the possibility that additional processes, such as attentional or environmental perturbation processes, may underlie the observed joint action effects. In addition, because most research has predominately focused on co- or shared-representation and action integration mechanisms, no previous research has attempted to examine the time-evolution or behavioral dynamics of actors’ responses during JSRC tasks. Consequently, it is possible that JSRC effects might be a result of the dynamical coupling processes that are known to exist during co-present joint-action situations [17], [18]. Indeed, the formation of shared representations may actually entail emergent, time-evolving coupling and dynamic inter-agent response modulation. The aims of the present study were therefore to (i) examine the dynamical structure of JSRC task behavior and (ii) investigate whether the standard (visual) JSRC effect might be a result of dynamic processes that couple and perturb the response behavior of co-acting individuals.

### Examining the Dynamics of JSRC

The crux of the traditional statistical analyses for JSRC experiments is a comparison of means, wherein each participant’s time series of responses is represented as a single, unchanging number. The average RT for each condition is understood as capturing the core and most meaningful aspect of the recorded RT behavior. The variability or time-evolution that occurs from trial to trial is simply discarded as error or mentioned only briefly in terms of how localized the mean is (for an exception see [19]). The temporal structure of RT variability (i.e., deviations from the mean over time), however, often provides additional and meaningful information about how behavior changes over time [20]. For instance, there is evidence that the seemingly error-induced variation in responses may actually be reflective of how people execute discrete motor responses in a certain spatiotemporal context [21]. Furthermore, even if the mean value and standard deviation are the same, the structure of RT time series that result in those means and standard deviations could in fact be quite different.

In order to examine the dynamic structure and unfolding variability of RTs over time, recent research has utilized fractal methods that provide deeper insight into the dynamics of an ongoing activity [22], [23], [24], [25]. Conceptually similar to geometric fractal patterns [26], fractal patterns in experimental time series data correspond to nested patterns of variability found across repeatedly-measured behaviors. Instead of comparing the overall means, fractal analysis determines how the variability exhibited in a time series changes with changes in time-scale. That is, fractal analysis determines if the structure of variability in an RT time series is statistically *self-similar* or *scale invariant*, such that small variations in the data have essentially the same structure as large variations [27], [28]. As in geometrical fractal patterns, if one were to “zoom in” (i.e., examine at a smaller scale) on the measurement time series, one would discover essentially the same pattern of fluctuations evident at the larger scale [29]. Accordingly, fractal statistical methods do not rely on partitioning the variability in measurement into different components, but rather assess the structure of the time-evolving variability observed.

A time series containing random fluctuations (i.e., white noise) indicates that the observed variability is the result of unsystematic or unrelated changes from one trial to the next [30]. Alternatively, the variability in an RT time series containing fractal or scale invariant structure contains trial-to-trial variability that is long-term correlated. In other words, the time series contains nested patterns of variability wherein small variations in measurement have the same structure as large variations. Such structure in repeated measurements is often referred to as ‘pink noise’ or 1/*f* noise, and is characteristic of a wide range of naturally occurring complex (interaction-dominant) systems and phenomena, from eye movement patterns [31] and heart rate variability [32], to self-reported mood change [33].

There are numerous methods for determining the degree to which the variability in a behavioral or response time series is *scale invariant* or pink (see [34] for a review). One of the most robust methods is the *detrended fluctuation analysis* (DFA; [22], [35]). DFA quantifies the long-term correlative properties of behavior by detrending the time series of adjacent bins, or collections of consecutive data points, at all time scales. The residual variance obtained from the least-square regression line subtraction of each bin is calculated for progressively larger bin sizes. Bin size is plotted against variance on a log-log plot, and the scaling exponent, *H,* is revealed by the slope of the best-fitting line. For DFA, *H* ≈ 1.0 indicates that the response variability or ‘noise’ is pink (i.e., fractal). White noise, however, corresponds to *H* = 0.5.

Deviations away from “perfect” pink noise (i.e., *H* = 1.0) are thought to reflect changes in system flexibility or constraint [36]. For instance, increasing task constraints or difficulty, such as coupling responses to a metronome, or increasing task speed, can whiten RT variability and result in *H* << 1.0 [37], [38], [39]. Changes in *H* across conditions thus reveal how differing task manipulations result in processes that interact or constrain each other, as well as influence the overall organizational processes that underlie a series of behavioral responses [30]. Accordingly, the question considered here was, does the co-presence of an actor during a JSRC task change the fractal structure of an individual’s RT behavior, and if so, how and why?

One possibility is that the behavior of individuals during joint-action conditions is subtly coupled and that this coupling acts to perturb the behavioral responses of the individuals involved. It is well known that the addition of small perturbations during task performance can whiten the time-unfolding behavior of human performance [36], [40], [41], [42]. When performing a stimulus-response compatibility task, a participant must coordinate a large set of intrinsic processes associated with perception and action to support their ability to respond. The transformation to a joint task modifies the task environment by adding an additional set of processes and events — those associated with the intrinsic perception-action dynamics of the second participant. The co-present materialization of these intrinsic perception-action dynamics almost ensures that they will become at least partially coupled [43]. This coupling would then pull each participant's intrinsic coordinative patterns away from their preferred states. The net result is a whiter signal, as compared with solo performance. Since there is measurable coupling, the relative whitening is evidence of a mutual perturbation of intrinsic dynamics that supports and accommodates joint coupling.

Given the significant body of research demonstrating that the behaviors of co-present individuals often become dynamically coupled (see [43], [44] for reviews) and that such coupling modulates and perturbs individual behavior (e.g., [45], [17], [46]), it would therefore seem likely that the fractal structure of the RT variability would be whiter (i.e., become less ‘pink’) in the joint condition compared to the individual condition. To explore this possibility, we employed a standard JSRC task, the Simon task [47], and had participants complete the task under joint and individual go/no-go conditions. We performed a fractal analysis on the resting RT time series using DFA, with the expectation that the joint condition would exhibit a whiter fractal structure (*H* closer to 0.5) compared to RT time series in the individual condition.

In addition to performing a fractal analysis, we also employed *instantaneous cross-correlation* [48] to index the degree to which the RTs of co-acting individuals were correlated (i.e., coordinated) with each other over time. If the behavioral responses of individuals are entrained during a joint-action situation, then the temporal correlation should be greater between the RT time series of individuals in the joint condition compared to RT time series of pseudo–pairs created using RT time series from participants who performed the task in the individual condition. The method is ideally suited for determining highly subtle non-synchronous coordination that occurs at variable time lags. It essentially computes the correspondence between two signals recursively, generating a time series of how past and future samples are correlated at all points in time. Setting a minimum *r^2^* value as a cut-off for what is considered to be correlated or not (i.e., *r^2^* = .25) then allows one to calculate the percentage of points that resulted in correlation values greater than that cutoff. The resultant value is the proportion of correlated activity and can be understood as providing a measure of percent coupling. The degree of correlated activity can also be indexed using instantaneous cross-correlation by determining the longest sequence of correlated activity (i.e., the longest sequence of sequential points/samples that remain above the minimum *r^2^* value) that occurs between the two time series. That is, the more correlated (temporally coupled) the two time series are, the longer the maximum line length (i.e. *maxline*).

## Materials and Methods

### Ethics Statement

The University of Cincinnati Institutional Review Board approved this study. Informed written consent was obtained from all participants prior to participation.

### Participants

Thirty-two undergraduate students from the University of Cincinnati (11 male, 21 female) participated in the study. They ranged in age from 18 to 22 years old and received class credit for participation in the experiment.

### Materials

A 19” Dell Flat Panel monitor was used to present stimuli. Stimuli included a blue “X” or red “X” (1″ high, ½″ wide), displayed on the left or right of the screen (positioned 5½″ from the top and bottom of the screen, and 2″ from the left or right side of the screen, respectively). Stimulus presentation and data collection were controlled using Direct RT. An Apple keyboard, modified to be millisecond accurate, was used to collect reaction time data. The shift keys were used as response indicators on the keyboard. A red sticker was placed on the right shift key and a blue sticker was placed on the left shift key. The monitor and the keyboard were placed in the center of a desk, with the keyboard 7″ from the front of the desk and 8″ from the monitor. Participants were seated in chairs that were placed next to each other in front of the keyboard. Each seated participant was positioned approximately 30″ from the display screen.

### Procedure

**Figure 1:**
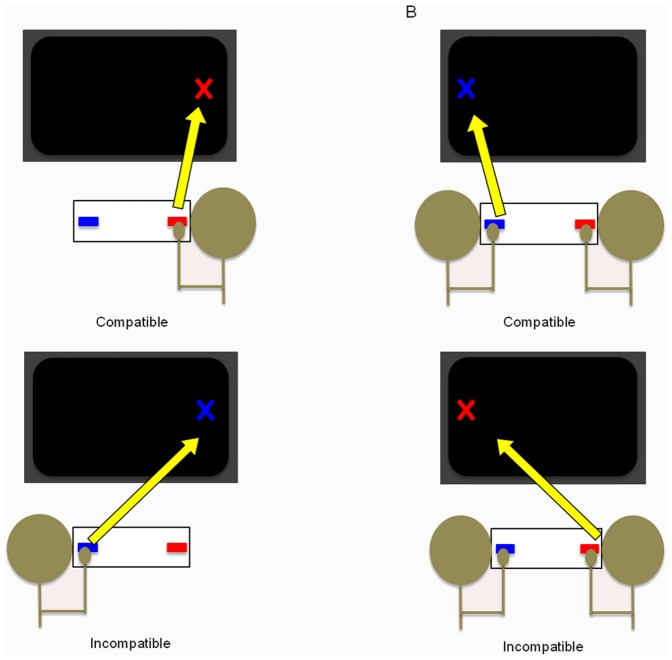
Experimental setup for (A) the individual condition, and (B) the joint condition.

Participants completed a visual go/no-go Simon task in which they were instructed to respond with a key press to a specific color of a stimulus presented on the screen. Participants were assigned only one of the two stimulus colors (e.g., red) and were instructed to respond only to their designated color, regardless of location, while ignoring the alternative color (e.g., blue). Participants completed the task in one of two experimental conditions: a joint condition or an individual condition. For the individual condition, participants performed the task alone. For the joint condition, pairs of participants performed the task together. Similar to the procedure of [6], subjects assigned to the red key sat on the right, and subjects assigned the blue key sat on the left, regardless of condition (see Figure 1). A brief instruction screen was presented on the computer monitor prior to the start of the experiment. Clarifying instructions were administered verbally and an opportunity for questions or clarification was offered.

Each trial began with a white crosshair presented for 400 ms in the center of the screen, followed by a blank screen also lasting 400 ms. Stimuli were presented for a maximum time of 1200 ms or until a response was indicated. Irrespective of RT, 400 ms of a blank screen was then presented 1200 ms after the stimulus presentation, followed by the white crosshair indicating the beginning of the next trial. In all conditions, participants completed 1100 trials, preceded by eight practice trials. An equal number of red and blue stimuli on both the left and right sides of the display were presented in a random order over the duration of the experiment.

## Results

### Analysis of Mean Reaction Time

**Figure 2:**
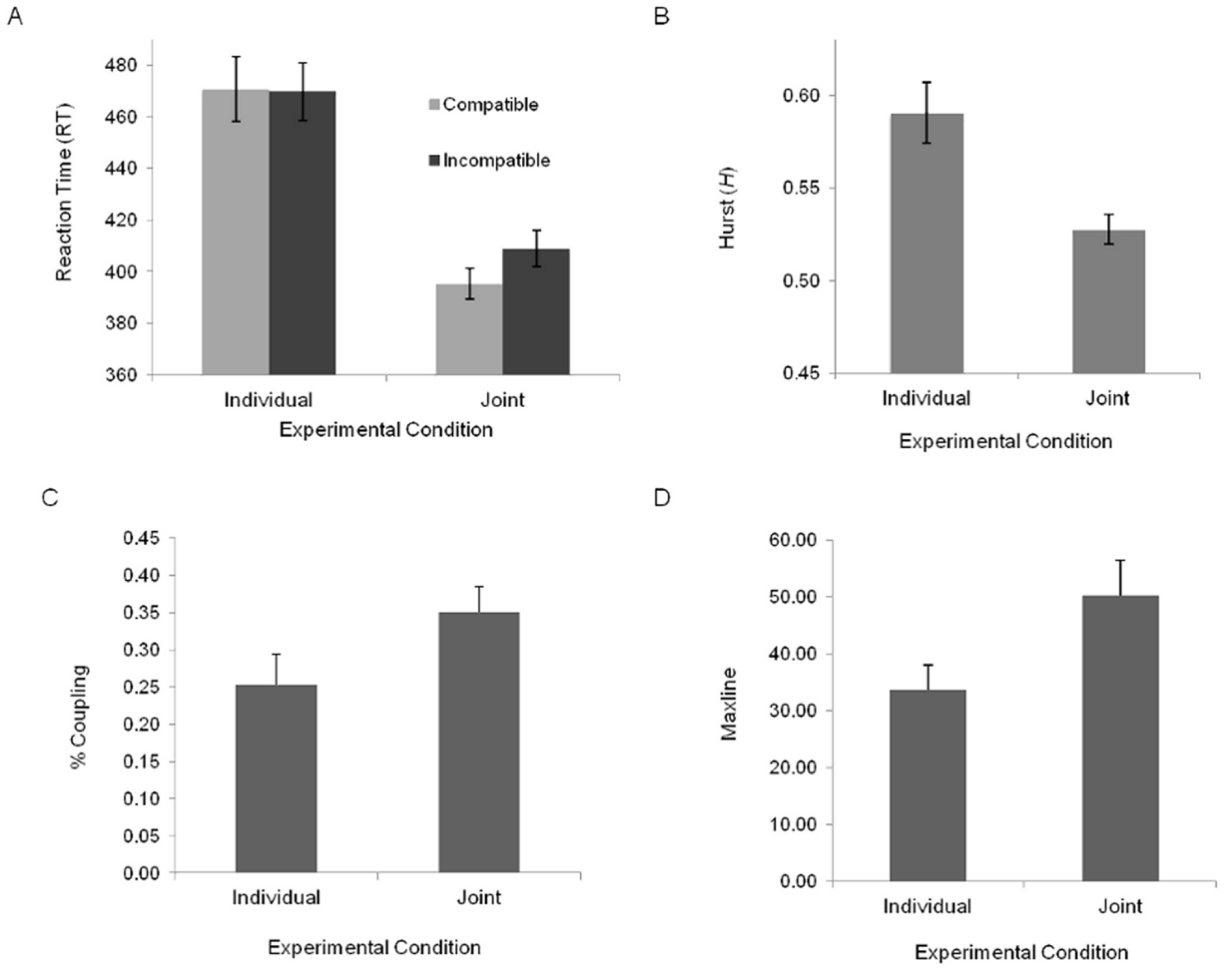
Results of linear and non-linear analyses. (A) Mean reaction time (RT) as a function of experimental condition and compatibility; (B) mean Hurst *(H)* as a function of experimental condition; (C) the percent coupling, (D) and maxline calculated using instantaneous cross correlation as a function of experimental condition.

A 2 (compatible vs. incompatible) × 2 (joint vs. individual experimental condition) mixed design analysis of variance (ANOVA) was conducted to determine whether the standard JSRC effect had occurred. Consistent with previous research [6], [7], the analysis revealed a significant interaction between response compatibility and experimental condition, *F*(1,30)  = 4.57, *p*<.05, with an effect of compatibility only being observed in the joint condition. This was confirmed using Bonferroni post hoc analyses, indicating that mean RTs were significantly faster for compatible responses (*M* = 395, *SD* = 33) than for incompatible responses (*M* = 409, *SD* = 40) in only the joint condition (*p*<.05). There was also a main effect of experimental condition, *F*(1,30)  = 12.90, *p*<.01, with RTs in the joint condition (*M* = 402, *SD* = 36) being significantly faster than RTs in the individual condition (*M* = 470, *SD* = 68) (see Figure 2A).

### Fractal Analysis

DFA was performed on the last 512 responses for each participant in the individual and joint condition. Prior to analysis, the RTs were normalized by subtracting the relevant condition means for each participant in order to examine the variability of the residual fluctuations (see [20] for a detailed description of the rationale). Consistent with our hypothesis that participants responding in the joint condition would exhibit a whiter fractal structure of responses due to task constraints and coupling, a between samples one-tailed *t*-test performed on *H* values calculated using DFA revealed a significant effect of experimental condition, *t*(30) = 9.71, *p*<.05, with the fractal structure of RTs in the joint condition being significantly lower *H* (*M* = 0.53, *SD* = 0.05) than in the individual condition (*M* = 0.56, *SD* = 0.10) (see Figure 2B). One-sample *t-*tests indicated that *H* values were significantly different from a test value of 0.5 (hypothetical white noise) for the individual condition, *t*(15)  = 5.12, *p*<.01. It should be noted that the current experimental design prevented a comparison of the structure of compatible versus incompatible responses, as dividing the RT behavior of participants into separate compatible and incompatible RT time-series can destroy the temporal structure dependence essential to fractal analysis.

### Instantaneous Correlation

Instantaneous cross-correlation allows one to determine how correlated two behavioral time series are across multiple time-scales. To determine the degree to which the RT time series of participants in the joint condition were entrained or coupled to each other over time, we calculated the percentage of correlations within the time series of instantaneous correlations for delays of −24 to 24 trials that had an *r^2^* >.25. As mentioned above, the resultant value can be understood as a measure of *percent coupling* or the proportion of correlated activity. We also determined the maximum line length, or *maxline*, of correlated activity above *r^2^* >.25. We then used between samples one-tailed *t*-tests to compare the percent coupling and maxline observed between participants in the joint condition to the percent coupling and maxline calculated between pseudo pairs of participants created by randomly pairing different participants from the individual condition. Consistent with the hypothesis that the behavioral responses of participants in the joint condition might be dynamically coupled, the analysis revealed that the percent coupling for the joint condition (0.35%) was significantly greater, *t*(30) =  −1.83, *p*<.039, compared to pseudo pairs (0.25%) created from participants in the individual condition (see Figure 2C). Similarly, the maxline for the joint condition (50.25) was significantly greater as compared to pseudo pairs (33.69) created from participants in the individual condition, *t*(30)  = −2.15, *p*<.02 (see Figure 2D). It is worth noting that, for validation purposes, we also conducted the instantaneous correlation analysis using delays of ±12, ±16, and ±32. The same pattern of results was observed across all of these delays. For all analyses, we employed a conservative *η* of.1 (see [44] for more details about this parameter).

## Discussion

The experimental study presented here was aimed at examining the behavioral dynamics of individuals during a joint-action stimulus-response compatibility task. We submitted recorded RT time series during a JSRC task to both a standard comparison of means, and to various dynamical analysis methods in order to examine how RT variability evolved over time. We compared these patterns of variability between joint and individual conditions.

Consistent with previous research, we found a significant difference in the overall reaction times between the individual and joint conditions, as well as a significant compatibility effect in the joint condition. More importantly, by measuring the fractal structure of participants’ RTs, we found that the structure of variability in the joint condition was much whiter than in the individual condition, as predicted. The current results therefore extend previous research by demonstrating that the mere presence of another individual not only affects average RT, but also affects the dynamics of an individual’s response behavior. This difference was theorized to be a consequence of an interpersonal coupling process that mutually perturbs the behavior of individuals in a shared environment [43].

To further examine whether the response behaviors of participants were dynamically coupled, an instantaneous correlation analysis was performed. We compared the degree to which the RT behavior of pairs in the joint condition was correlated to the degree of RT correlations that occurred for pseudo pairs created from participants who completed the individual condition. The results of this analysis revealed that the response behavior of pairs in the joint condition exhibited greater temporal correlation compared to pseudo pairs, providing more evidence that the response behaviors of co-present individuals in the current go/no-go task dynamically influenced each other. The results of examining changes in the temporal periods of correlated activity indicate the possibility that the presence of another individual acting as a perturbation influences the degree of response correlation over time. The magnitude of these temporal correlations was by no means large and occurred at non-synchronous time lags. The weak and complex nature of the interpersonal influence should not be discounted, however, given the fact that the mean differences in RT are also relatively small (as is typically the case in JSRC studies). Indeed, the relative change in mean RT, fractal dimension (*H*), and % coupling and maxline are all somewhat equivalent. Furthermore, like most other forms of interpersonal entrainment (see e.g., [49], [43] for reviews), the coupling that occurred was most likely irregular, rather than constant, and did not occur synchronously or at any fixed time lag. In other words, the influence of another person’s presence likely acted as an intermittent perturbation to an individual’s response behavior, resulting in non-continuous coupling and a whiter structure of variability.

It should be noted that it is possible that the difference in the average speed of responses in the individual and joint conditions could have contributed to the observed changes in the fractal scaling of individual and joint performance [36] – although it is hard to understand why or how a change in response speed could increase the instantaneous cross-correlation statistics of % coupling and maxline. Accordingly, a great deal more research is needed to fully understand how and why responding with another individual changes the fractal structure of response behavior, and the degree to which such effects are a manifestation of heightened activity due to the mere presence of another individual.

Finally, if one accepts that the differences in fractal structure and temporal correlation observed between the joint and individual conditions in the current study are due to interpersonal perturbation processes, the question becomes how or why such coupling should result in a compatibility effect (i.e., a discrepancy between reaction times on compatible versus incompatible trials). Unfortunately, the current design did not allow us to directly compare the structure of compatible and incompatible trials and, thus, the degree to which the current results can speak directly to this question is limited. Nevertheless, the current results are consistent with the possibility that co-acting individuals form a single synergistic animal-environment system, whereby the ongoing response behavior of each individual is functionally linked in a nonlinear (non-additive) manner to the present and previous activity of their co-actor and other environmental task constraints [47]. In support of this possibility, recent research has demonstrated that similar stimulus-response compatibility effects can emerge when a co-acting person is replaced with an object possessing some dynamic event-based quality [16]. It is plausible, therefore, that attunement to the location of a dynamical object changes the task space, and, consequently, provides additional constraints that affect the motor assembly required to make a response. Together with recent neuroscience research suggesting that planning events in the environment entails the same neural dynamics as detecting events in the environment [i.e., 50, 51], this suggests that the behavioral effects typically attributed to joint-action co-representation may merely reflect perturbations to the stable temporal and spatial state of an individual actor by other task-situated dynamic events (including other individuals). In other words, by simply sharing the same behavioral space with another dynamical element, individuals are no longer free to behave in an isolated manner, with interpersonal response modulation emerging as a natural consequence of being unintentionally bound within a task-specific synergistic animal-environment system.

In conclusion, the current study provides the preliminary evidence that the response behavior of co-actors during a JSRC task is dynamically coupled and that dynamical processes operate to constrain and perturb the time-evolving response variability of co-acting individuals. Although not directly tested here, it is possible that these dynamic processes of constraints may decouple behavior over time, and may therefore underlie the JSRC effect, rather than some form of shared representation. It is worth noting, however, that the dynamical systems and co-representational accounts of such behavior are not mutually exclusive and may in fact provide complementary explanations for such joint-action phenomena. Indeed, future research should be directed towards more extensive investigation of how intermittent entrainment could lead to enhanced representational correspondence between actors, and how such an alignment influences reaction time. By examining these issues further, future research may lead to a better understanding of how the dynamics of joint-action activity both shape and are shaped by joint cognitive or representational processes.
